# Meta-analysis of the optimal needle length and decompression site for tension pneumothorax and consensus recommendations on current ATLS and ETC guidelines

**DOI:** 10.1186/s13017-025-00613-7

**Published:** 2025-05-19

**Authors:** Suhaib J. S. Ahmad, Jason R. Degiannis, Marion Head, Ahmed R. Ahmed, Edgar Gelber, Sherif Hakky, Armin Kieser, Martin Müller, John Darling, Dominik A. Jakob, Ioannis Panagiotis Kyriazidis, Konstantinos Degiannis, Patrick Dorn, Anil Lala, Christopher Bowman, Danielle Wilkinson, Graham Whiteley, Umair Hassan, Younis Mohamed, Kai Hui Loo, Ynyr Dewi Davies, Richard Egan, Sjaak Pouwels, Amber Coulthard, Lowri Churchill, Kiran Bhavra, Christopher Bailey, Ian Johnson, Ifan Rees, Dafydd Williams, Shahab Hajibandeh, Wah Yang, Christian Peter Subbe, Amy Owen, David Rawaf, Ameer Khamise, Ali Waleed Khalid, Chetan Parmar, J. Agustin Soler, Miriam Khalil, Ata Mohajer-Bastami, Sarah Moin, Rami Archid, Mohamed Abdulmajed, Rosalind Jones, Vignesh Balasubaramaniam, Rawa Al-Salihi, Arran Shoker, Mei-Ju Hwang, Olga Griffiths, Sushil Pandey, Lucy Lee-Smith, Aristomenis K. Exadaktylos

**Affiliations:** 1 Department of General Surgery, Betsi Cadwaladr University Health Board, Bangor, Wales, UK; 2 Department of Emergency Medicine, Inselspital University Hospital of Bern, Bern, Switzerland; 3 Department of Neurosurgery, University Hospital Saarland, University of Saarland, Homburg, Germany; 4 Department of Surgery, Imperial College London, London, UK; 5 Department of Trauma and Orthopaedics, Betsi Cadwaladr University Health Board, Bangor, Wales, UK; 6 Department of Diagnostic, Interventional and Paediatric Radiology, Inselspital University Hospital of Bern, Bern, Switzerland; 7 Center for Musculoskeletal Surgery, Charité-University Hospital Berlin, Berlin, Germany; 8 Department of Thoracic Surgery, Inselspital, University Hospital of Bern, Bern, Switzerland; 9 School of Surgery, Health Education and Improvement Wales, Cardiff, Wales, UK; 10 Department of Surgery, Morriston Hospital, Swansea, Wales; 11 Department of Surgery, Klinikum Lippe, Bielefeld University- Campus Lippe, Detmold, NRW Germany; 12 Department of Intensive Care Medicine, Elisabeth-Tweesteden Hospital, Tilburg, The Netherlands; 13 Department of Anaesthesia & Intensive Care, Betsi Cadwaladr University Health Board, Bangor, Wales, UK; 14 Department of General Surgery, Morriston Hospital, Swansea, UK; 15 Department of Surgery, The First Affiliated Hospital of Jinan University, Guangzhou, China; 16 Department of Medicine, Betsi Cadwaladr University Health Board, Bangor, Wales, UK; 17 Department of Emergency Medicine, Betsi Cadwaladr University Health Board, Bangor, Wales, UK; 18 WHO Collaborating Centre for Public Health Education and Training, Imperial College, London, UK; 19 School of Medicine, University of Buckingham, Buckingham, UK; 20 Department of Surgery, Whittington Hospital, London, UK; 21 University College London, London, UK; 22 St James’s University Hospital, Leeds Teaching Hospital Trust, Leeds, LS9 7 TF UK; 23 Public Health Lead, Brompton PCN, Lonodon, UK; 24 East Surrey Hospital, Redhill, Surrey UK; 25 Department of General, Visceral and Bariatric Surgery, Diakonie-Klinikum Stuttgart, Teaching Hospital of the University of Tübingen, Tübingen, Germany; 26 Department of Urology, Mersey and West Lancashire Teaching Hospitals NHS Trust, Liverpool, UK; 27 Department of Obstetrics and Gynaecology, Betsi Cadwaladr University Health Board, Bangor, Wales, UK

**Keywords:** Tension pneumothorax, Needle decompression, Chest wall thickness, Needle length, Intercostal space, Iatrogenic injury, Trauma care

## Abstract

**Background:**

Tension pneumothorax (TP) is a life-threatening condition. The immediate recommended management is needle decompression (ND), followed by the insertion of an intercostal chest drain. The European Trauma Course (ETC) and the Advanced Trauma Life Support (ATLS) guidelines differ on needle size and decompression site, creating clinical uncertainty. This meta-analysis aims to explore the optimal approach for emergency needle decompression in TP.

**Methods:**

This meta-analysis followed the PRISMA 2020 guidelines. It included English-language RCTs, cohort, case–control, cross-sectional studies, and case series with more than six patients. Studies on adults undergoing needle decompression therapy for TP or with chest wall thickness measurements were included. Ovid MEDLINE, Embase, and Web of Science databases were searched until May 31, 2024. Data were extracted, assessed for quality using OCEBM and GRADE, and analyzed using SPSS and OpenMeta with random-effects models. Primary outcome: needle decompression failure rate. Secondary outcomes: patient demographics, cannula size, and chest wall thickness comparisons.

**Results:**

This review analyzed 51 studies on needle decompression for TP, with a weighted mean patient age of 36.67 years. Radiological data from 24 studies (n = 8046) indicated a 32.84% failure rate for needle penetration into the pleural cavity (I^2^: 99.72%). Increased needle length reduced failure rates by 7.76% per cm. No significant differences in chest wall thickness between genders were observed (T-test, p = 0.77), but thickness at the 5th anterior axillary line (5AAL) and 5th midaxillary line (5MAL) was less than at the 2nd midclavicular line (2MCL). Injury rates were higher at 5AAL than 5MAL, with strong positive correlations between needle length and injury at these sites (0.88, 0.91).

**Conclusion:**

Based on our meta-analysis, a 7 cm needle may be appropriate for decompression of right-sided tension pneumothorax at either the 5th intercostal space along the midaxillary line or the 2nd intercostal space along the midclavicular line. For left-sided cases, given the potential risk of cardiac injury, the 2nd midclavicular line is a safer option. However, these recommendations should be interpreted with caution due to considerable heterogeneity among the included studies, potential risk of bias, and variability in measurement techniques. Clinical decisions should always be individualized, taking into account patient-specific factors.

**Supplementary Information:**

The online version contains supplementary material available at 10.1186/s13017-025-00613-7.

## Introduction

The lung moves smoothly within the chest cavity due to the presence of two pleural layers, the parietal and visceral pleurae. Between these two layers is the pleural ‘cavity’, a potential space which contains a small amount (5–10) of pleural fluid and maintains a pressure of −4 mmHg at rest. The accumulation of air into the pleural cavity is a pneumothorax and leads to a degree of lung collapse [1]. Causes include trauma, medical intervention, primary spontaneous pneumothorax (no history of underlying lung disease), and secondary spontaneous pneumothorax (history of underlying pulmonary disease) [2]. A simple pneumothorax can develop into a tension pneumothorax (TP) if a valve mechanism forms, allowing air to enter the pleural space but preventing it from escaping [3]. This is a life-threatening condition requiring urgent intervention: the progressive increase in intrathoracic pressure displaces the mediastinum towards the opposite side, leading to reduced venous return to the heart and resulting in hemodynamic instability [4–6]. Immediate needle decompression (ND) is the first line of treatment, aiming to convert the tension into a simple pneumothorax. This is followed by the insertion of an intercostal chest drain as the definitive treatment [3, 7].

Recommendations from the European Trauma Course (ETC) and the Advanced Trauma Life Support (ATLS) regarding best practice for needle decompression in TP are contradictory. The ETC suggests using a 14 or 16-gauge “extra-long” cannula at the 2nd intercostal space along the midclavicular line (MCL), while the ATLS recommends a 5 cm for small adults or 8 cm needle for larger adults, at the 4 th or 5 th intercostal space in the anterior midaxillary line (MAL). This inconsistency is unhelpful, creating ambiguity for clinicians performing emergency needle decompression in TP, when time and precision are critical [5, 8–13]. In addition to addressing the ongoing debate regarding optimal needle length and insertion site, there is a critical need for standardized approaches to measuring chest wall thickness (CWT) and for clearly defined clinical outcome metrics. Moreover, current literature predominantly emphasizes short-term results, offering limited insight into long-term complications such as iatrogenic injuries or the need for repeat interventions. This meta-analysis not only seeks to evaluate procedural efficacy but also aims to underscore these important gaps in the existing evidence base.

Accordingly, we undertook this meta-analysis to systematically review the available data and provide evidence-based recommendations on appropriate needle length and decompression site selection.

### Patients and methods

This systematic review and meta-analysis strictly adhered to the guidelines of the Preferred Reporting Items for Systematic Reviews and Meta-analyses (PRISMA) 2020 [14].

### Inclusion criteria

Types of Studies:

- English language
- Randomized Controlled Trials (RCTs)

Prospective and retrospective observational studies:

- Cohort studies
- Case–control studies
- Cross-sectional studies
- Case-series with more than 6 patients

Population:

- Adults (> 18 years) undergoing Needle Decompression Thoracostomy (NDT) for proven or suspected TP OR adults who had their chest wall thickness (CWT) measured by ultrasound, computed tomography (CT), or magnetic resonance imaging (MRI).

### Exclusion criteria

Types of Studies:

- Animal studies
- In vitro studies
- Case reports
- Commentaries
- Review studies
- Conference proceedings

Procedures and Treatments:

- Studies where needle aspiration and not decompression was performed as first stage management of TP

### Information sources

Ovid MEDLINE, Embase, and Web of Science, from 1946 to May 31 st, 2024.

### Search strategy

A detailed search strategy in Supplementary Table 1a and 1b, performed by three independent reviewers.

### Selection process

Screening of titles and abstracts, followed by full-text reviews against criteria, with discrepancies resolved through discussion with a third reviewer.

### Data collection process

Standardized form data extraction by two independent reviewers (SJSA, JRD, MH).

### Data extraction

Data extracted are presented in supplementary Table 2.

### Quality assessment

Quality assessment conducted by three independent investigators (SJSA, JRD, MH, ARA, SP) using the Oxford Centre for Evidence-Based Medicine (OCEBM) Levels of Evidence and the GRADE system, with discrepancies resolved through consensus.

### Statistical analysis

The statistical analysis was conducted using SPSS (Chicago, IL, USA, Version 20.0) and OpenMeta. These software packages facilitated the random-effects meta-analysis, heterogeneity assessment, publication bias assessment through funnel plots and Egger's test, and additional sensitivity analyses [15]. Effect sizes (mean difference, odds ratio etc.) and results of meta-analysis will be presented with 95% Confidence Interval (CI).

A 2-sided P < 0.05 will be considered statistically significant.

### Primary outcome

Needle decompression thoracostomy (NDT) failure rate, in TP, estimated via a random-effects meta-analysis model.

### Secondary outcomes

Patient Demographics:

- Age, gender, and body mass index (BMI)

Cannula Size:

- Comparison of different lengths.

Chest Wall Thickness:

- Comparison of chest wall thickness at various decompression sites—including the midclavicular line (MCL), midaxillary line (MAL), and anterior axillary line (AAL)—as assessed using ultrasound (US), computed tomography (CT), or magnetic resonance imaging (MRI).

### Complication rate analysis

- Detailed analysis of complications associated with needle decompression thoracostomy (NDT) procedures.

### Synthesis methods

Employed Der Simonian and Laird method for random-effects meta-analysis to estimate pooled failure rates, assessing heterogeneity with the I^2^ statistic [16].

### Reporting bias assessment

Funnel plots and Egger's test for publication bias assessment, considering small study effects. The ROBINS-I was used for Non-randomized controlled trials [17].

### Certainty assessment

The GRADE approach evaluated evidence certainty, considering study limitations, inconsistency, indirectness, imprecision, and publication bias.

### Definition of clinical success of needle decompression

Needle Thoracostomy effectiveness was categorised as having a positive clinical response if any of the below identified clinical improvement criteria occurred [18]:

· Relief of Respiratory Distress
· Improvement in Hemodynamic Stability
· Decreased Jugular Venous Distension (JVD)
· Improvement in Oxygen Saturation
· Return of Respiratory Breath Sounds
· Decrease in Tracheal Deviation
· Decreased peak inspiratory pressure

It is important to note that different studies used varying criteria or combinations thereof, leading to a lack of uniformity in how clinical success was defined, potentially affecting comparability.

## Results

**Fig. 1:**
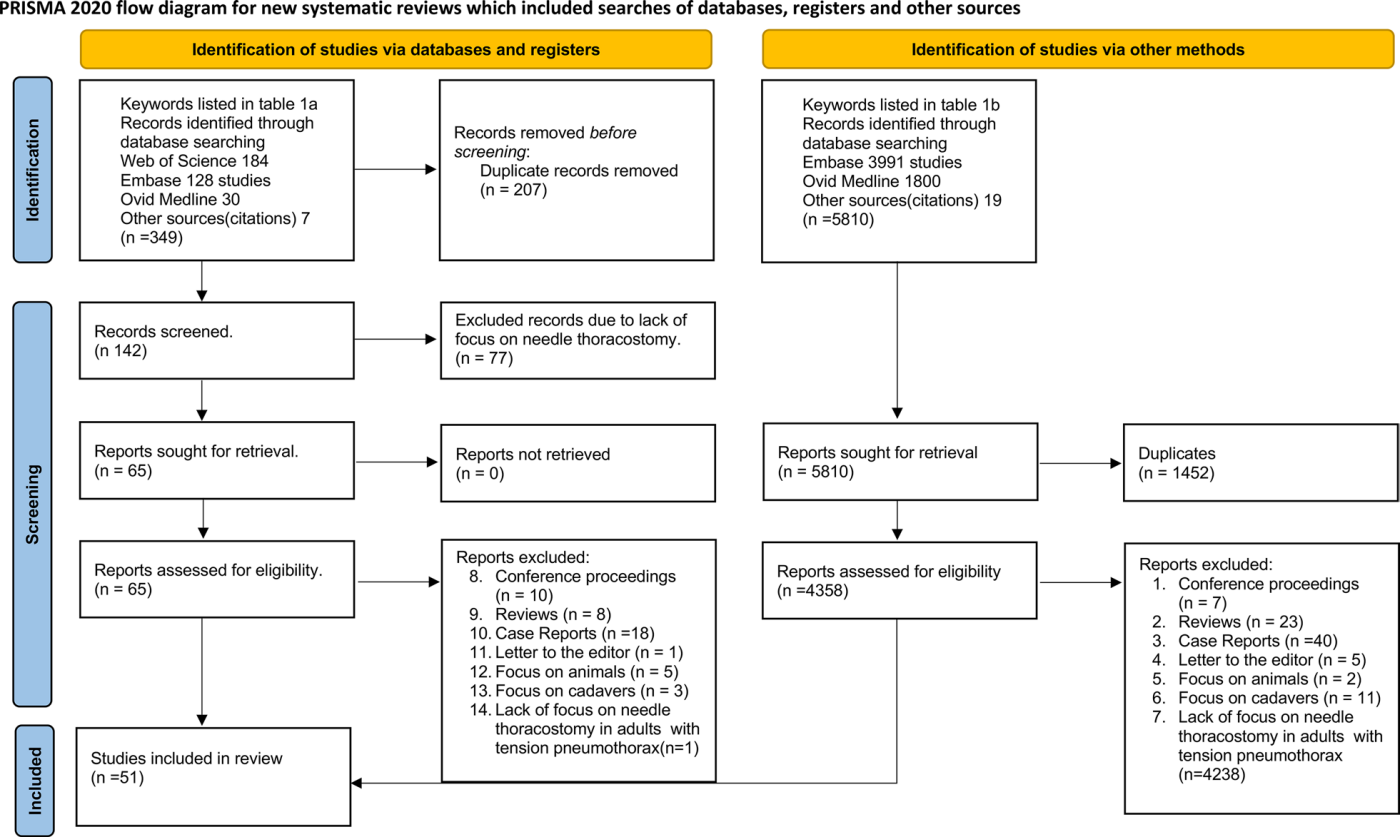
PRISMA flow diagram

The detailed data extraction is illustrated in the PRISMA flow diagram as shown in Fig. 1.

Supplementary Table 3 presents the fifty-one publications included in the study.

Out of the 51 studies analysed, 12 were prospective and 39 retrospective. There were 46 case-series, 3 cross-sectional studies, 1 case–control and 1 cohort study.

In terms of the evidence hierarchy as per the OCEBM standards, 50 articles fell into Level IV evidence and 1 was categorized as Level III. The Egger test showed an intercept value of 8.35, a slope of 0.08 and a P-value of 0.999. These values indicate no significant evidence of publication bias in this dataset. The high p-value suggests that the observed effects are likely not due to bias in the published studies.

**Fig. 2:**
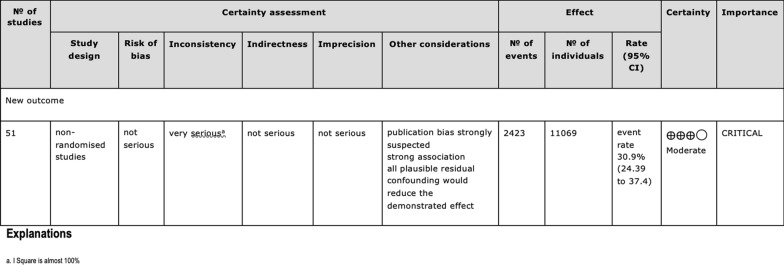
The GRADE tool assessment of the 51 included studies

The GRADE tool showed that all 51 studies were of moderate certainty of evidence (Fig. 2).

**Fig. 3:**
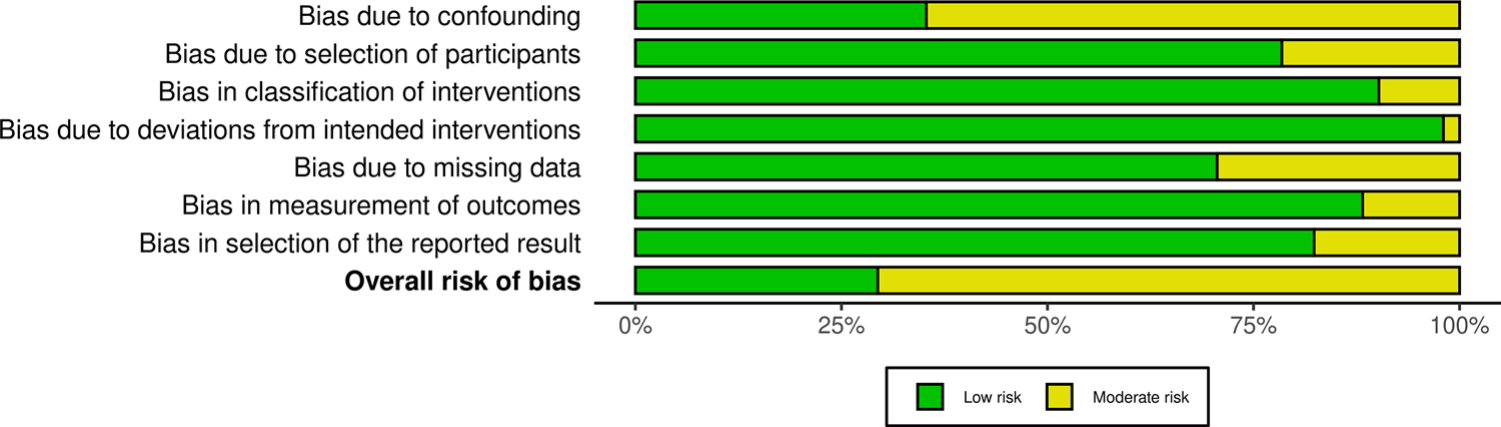
Summary of the risk of bias

Using the Risk of Bias in Non-randomized Studies (ROBINS-I) tool, 36 of the 51 articles were classified as having a moderate risk of bias, while 15 studies were low risk. Figure 3 provides a detailed summary of the bias risk assessment as evaluated by the ROBINS-I Tool.

The weighted mean age of patients included in this study was 36.67 years (CI 25.73–47.61). The total number of patients who sustained a pneumothorax was 11,069, of whom approximately 2423 (67% male) underwent needle decompression thoracostomies; in some cases, more than one attempt was required. Radiological findings were reported in 24 studies, encompassing a total of 8,046 patients.

Approximately 82% of the injuries were related to blunt trauma and approximately18% to penetrating trauma. Twenty studies reported their experience in pre-hospital, while 33 articles reported decompression in a hospital setting [11, 18–67].

### Radiological chest wall thickness

Supplementary Table 4 shows the average CWT and range in males and females at three different decompression sites (2MCL, 5 AAL and 5MAL).

The T-test indicated that there was no significant difference between male and female CWT measurement statistics: −0.29, P-value: 0.77.

For both males and females, the Tukey HSD post-hoc test indicated that the CWT at 5 AAL and 5MAL is less than at 2MCL. Furthermore, there was a significant difference in CWT between the 5MAL and 5 AAL sites:

CWT at the 5 AAL site is significantly thinner than at the 5MAL site (P < 0.05).

The mean CWT varied across countries but showed no statistically significant differences according to ANOVA tests (Supplementary Table 5).

The regression analysis indicates that there is a positive, weak and statistically insignificant relationship between BMI and average CWT Slope: 0.4436, P-value: 0.7195.

### Overall failure rate of needle decompression in tension pneumothorax

**Fig. 4:**
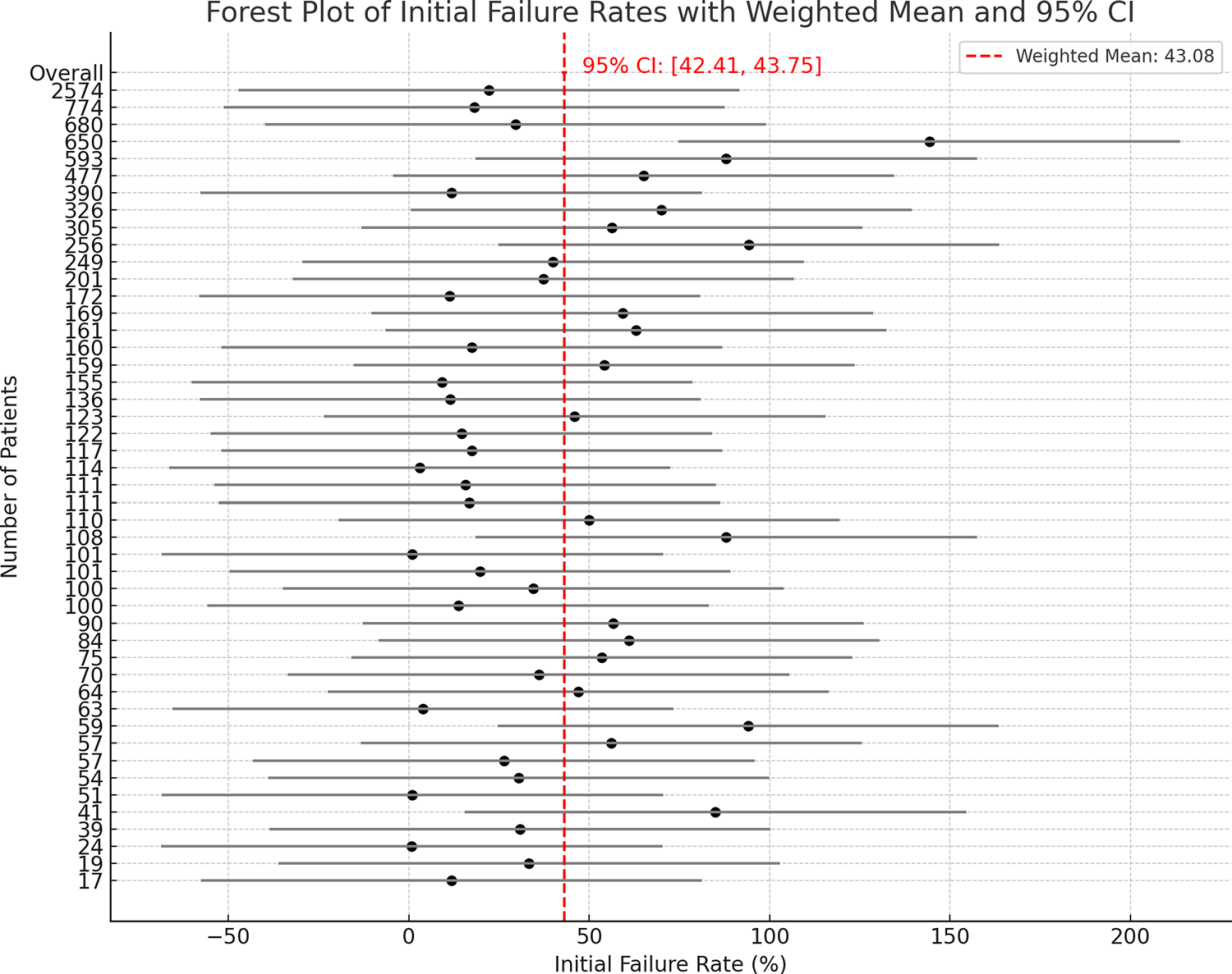
Forest plot of the initial failure rates of needle decompression in tension pneumothorax

The overall combined weighted average failure rate—clinical and radiological—was approximately 43.08% (95% confidence interval [CI]: 42.41–43.75) (Fig. 4). These figures represent averages across highly heterogeneous studies (I^2^ > 99%) with differing patient populations, imaging modalities, and outcome definitions, all of which limit the generalizability and direct comparability of our findings.

### Clinical failure rate based on symptom improvement

**Fig. 5:**
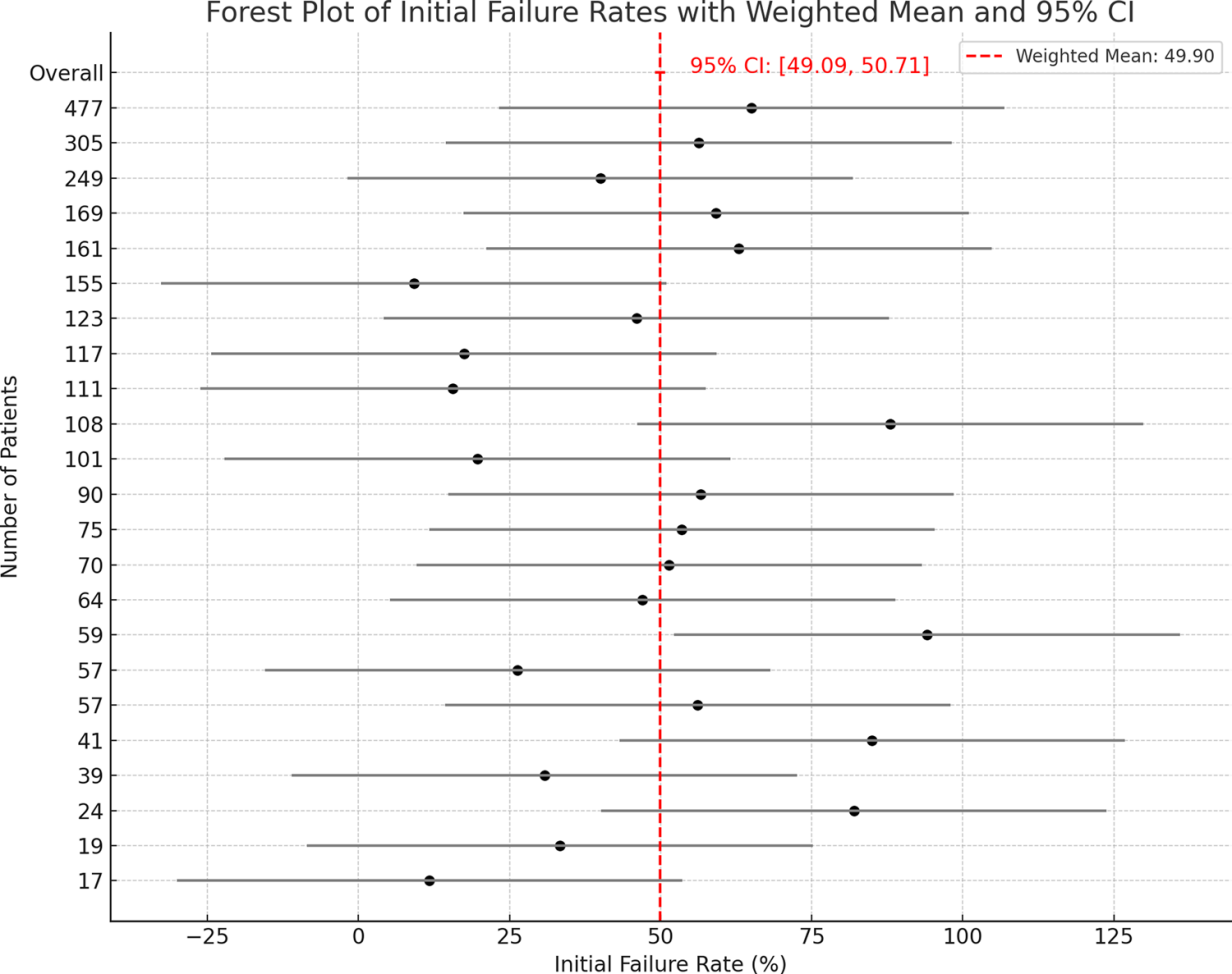
Forest plot of clinical failure rate based on symptom improvement

When looking specifically at the failure rate for achieving clinical improvement (meaning a noticeable positive change in the patient’s condition), it was 49.90% (CI 49.09–50.71). The I^2^ = 99.18% (Fig. 5).

### Clinical failure rate related to needle length and chest wall thickness as determined by CT, MRI, or ultrasound

**Fig. 6:**
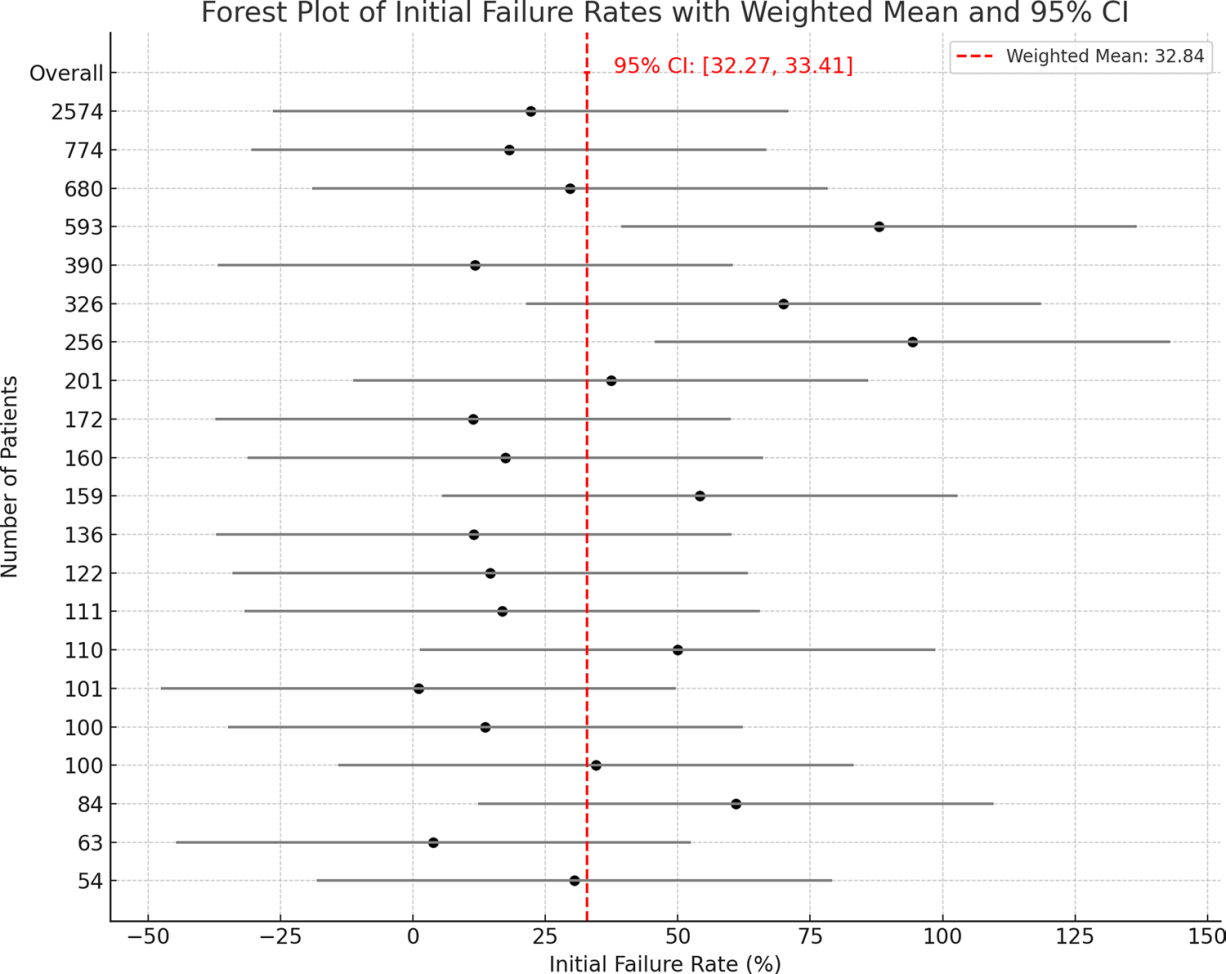
Forest plot of clinical failure rate related to needle length and chest wall thickness

For the proportion of cases where the needle successfully reached the pleural cavity after penetrating the chest wall, as assessed using radiological imaging, at different sites and needle lengths, the failure rate was 32.84% (CI 32.27–33.41), with an I^2^ value of 99.72% (Fig. 6).

### Assessing radiological failure rates (US/CT/MRI) of different needle lengths at different sites

**Fig. 7:**
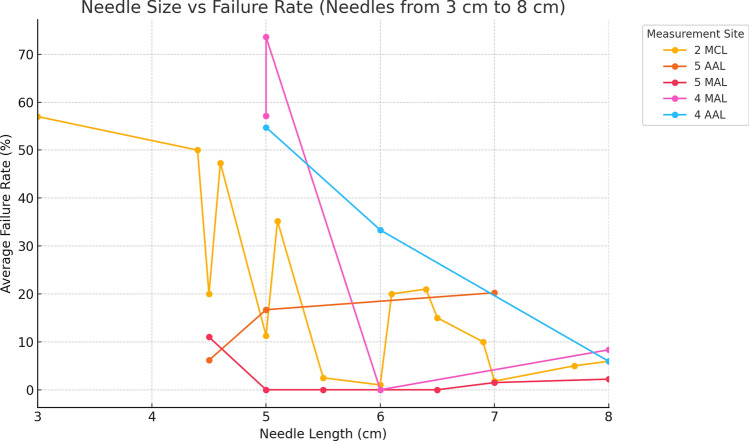
Needle size vs failure rate at different sites (Needles from 3cm to 8cm)

There is a statistically significant negative effect of increasing needle length on failure rates for entering the pleural space. As needle length increases, the failure rate tends to decrease. Figure 7, Supplementary Table 6a,b,c

### Overall trend

- For every 1 cm increase in needle length, the failure rate decreases by approximately 7.76 % points, on average.
- **Intercept**: 65.43
- **R-Squared**: 0.317
- **p-value**: 0.00064

The ANOVA results for failure rates across the sites, 2MCL, 5 AAL and 5MAL, standardized by needle length, are:

- **F-statistic**: 2.90
- **p-value**: 0.0516

The p-value (0.0516) is above the typical significance threshold of 0.05, indicating that there is no statistically significant difference in failure rates between sites when comparing standardized needle lengths, but the result is noted to be close to significance.

### Potential injury to the heart on insertion of needle thoracostomy on the left side of the thorax. Figure 8

**Fig. 8:**
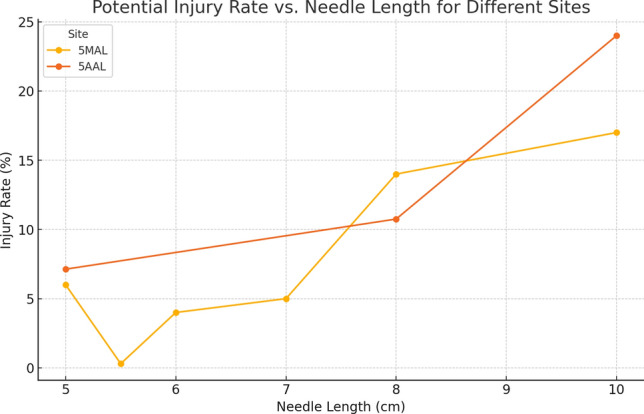
Potential injury vs needle length for different sites at different sites

The Pearson correlation coefficients, which quantify the strength of this relationship, are as follows:

- **Left 5 AAL: **0.88 (strong positive correlation)

- **Left 5MAL: **0.91 (strong positive correlation) 

## Discussion:

The primary goal of needle thoracostomy is to convert a TP into a simple pneumothorax, followed by definitive management via the insertion of an intercostal drain [9]. Diagnosing a TP in emergency settings can be challenging, especially when key clinical signs such as reduced/absent breath sounds, tympanic percussion, or tracheal deviation are difficult to elicit [3–5]. Inserting a needle into the thoracic cavity in cases of misdiagnosed TP can lead to iatrogenic pneumothorax, complicating subsequent treatment [10].

Patient-specific factors such as anatomical variation, trauma mechanism, chest wall compliance, and comorbidities (e.g., obesity, chronic pulmonary disease) must be taken into consideration, when choosing site of needle decompression and the needle length. Due to that, there are different recommendations related to the presence or absence of the above factors. It is true that these recommendations serve Health professionals and their patients well. On the other hand, it is important to follow some standard recommendations that will apply to all type of patients and will lead to swift and successful management of TP. This has been attempted by the ATLS and ETC organisations but unfortunately, there is no consensus between the two, leading to possible confusion.

The aim of our meta-analysis is to bridge the gap between the above, by recommending a standardised site for decompression as well as length of the decompression needle irrespective of the presence or absence of what is nowadays considered confounding factors.

Our meta-analysis indicates that, on average, increasing needle length is associated with a reduction in failure rates. However, Specifically, for each additional centimeter of needle length, the failure rate decreases by approximately 7.76 percentage points. This finding is important in light of the conflicting recommendations from the European Trauma Course (ETC) and Advanced Trauma Life Support (ATLS) guidelines. The ETC recommends a 14- or 16-gauge “extra-long” cannula, while ATLS suggests using a 5 to 8 cm needle depending on patient size [5, 7, 10–13], leading to confusion and potential delay during emergency situations where precision and timeliness are critical.

No significant difference in CWT was observed between male and female patients (T-test statistic: −0.29, p = 0.77), which suggests that factors such as BMI may be more important in determining appropriate needle length. The chest wall was shown to be consistently thinner at the 5 th anterior axillary line (5 AAL) compared to the 2nd MCL (p < 0.05), supporting the preference for certain sites depending on the patient’s body build and trauma type. Interestingly, our regression analysis demonstrated no strong relationship between BMI and CWT (slope: 0.4436, p = 0.7195), which may be explained by BMI’s inability to account for fat distribution and muscle mass differences that can influence CWT.

The risk of heart injury during left-sided decompression is a significant concern, particularly with longer needles. TP displaces the mediastinum, but this effect is more pronounced in the upper mediastinum than in the lower, leaving the heart vulnerable to injury, especially if the needle is inserted at the 5 th ICS. Our study showed a strong correlation between needle length and injury risk at the left 5 AAL and 5MAL sites was strong, with increasing needle length associated with higher injury rates (correlations of 0.88 and 0.91, respectively). Therefore, we suggest that the 2nd ICS along the MCL may be a safer site for left-sided decompression to reduce this risk, but this should be guided by clinical context and patient anatomy.

Compared to previous literature, our findings support the use of longer needles, which several studies have found to be more effective at successfully penetrating the pleural cavity. Hecker et al. [38] demonstrated that a 7 cm needle was successful in over 90% of cases when inserted at the 2nd MCL, which aligns with our recommendations. Other studies have similarly concluded that shorter needles, particularly 5 cm, often fail to penetrate the pleural space, particularly in patients with a higher BMI or a thicker chest wall [37, 49]. However, although increasing needle length improves success rates, it also results in increased risk of injury to underlying structures [30, 45, 56, 60], thus requiring careful consideration during clinical decision-making.

## Limitations

This meta-analysis has several limitations that must be considered. Firstly, the included studies showed a high level of heterogeneity (I^2^ > 99%) in terms of design, patient demographics, and trauma settings, which limits the generalizability of our findings [16]. Secondly, most studies were retrospective and fell into lower evidence levels (Level IV), with only one study at Level III, potentially introducing bias. The use of different imaging modalities (ultrasound, CT, MRI) introduces variability in chest wall thickness measurements, particularly since ultrasound is operator-dependent and can be influenced by factors such as probe pressure and tissue compressibility [27, 28]. The regression analysis on BMI and chest wall thickness, though included, yielded weak and statistically insignificant results, suggesting BMI may not be a reliable predictor for CWT [30, 45, 56, 60].

Furthermore, clinical improvement criteria varied significantly between studies, ranging from oxygen saturation improvement to more subjective measures like breath sound return. This inconsistency reduces the comparability of results.

Additionally, most included studies focused on immediate or short-term outcomes. Data on long-term complications—such as persistent iatrogenic injury, infection, or repeated interventions—were scarce. Future research should aim to address these gaps, ideally through prospective, multi-centre trials using standardized techniques and outcome definitions [68–77].

## Conclusion

- The purpose of this study is to facilitate effective decompression of tension pneumothorax irrespective of the individual chest wall, by recommending a standardized decompression site and needle length. Based on our meta-analysis, we suggest the following:
- Right-sided tension pneumothorax, should be decompressed with a 7 cm decompression needle, inserted at the 2nd intercostal midclavicular line or the 5 th midaxillary line.
- Left-sided tension pneumothorax, should be decompressed with a 7 cm decompression needle, inserted at the 2nd intercostal midclavicular line.
- Left-sided tension pneumothorax should not be decompressed at the 5 th midaxillary line, due to risk of cardiac injury.
- We believe that the above is a viable proposition. However, taking into consideration the limitations of this study, we feel that it is of paramount importance for its suggestions to be assessed by prospective, multi-centre trials.

## Supplementary Information

Additional file 1.

## Data Availability

No datasets were generated or analysed during the current study.

## Declarations

### Patients and public involvement

No patients were involved in this study as it is a meta-analysis.
