# Supplementary material for: Meta-analysis of the optimal needle length and decompression site for tension pneumothorax and consensus recommendations on current ATLS and ETC guidelines
Source: World J Emerg Surg. 2025 May 19;20:39. doi: 10.1186/s13017-025-00613-7 (PMC12087068; doi:10.1186/s13017-025-00613-7)
Supplement: Supplementary file 1 — Additional file 1. [file 13017_2025_613_MOESM1_ESM.docx]

**Supplementary**

**Supplementary Table 1a:** Search strategy

| **Database: Ovid MEDLINE(R) ALL <1946 to April 30, 2024>**  **Search Strategy:**  **1**  needle decompression.mp. (238)  **2**  (needle adj2 decompression).mp. [mp=title, book title, abstract, original title, name of substance word, subject heading word, floating sub-heading word, keyword heading word, organism supplementary concept word, protocol supplementary concept word, rare disease supplementary concept word, unique identifier, synonyms, population supplementary concept word, anatomy supplementary concept word] (262)  **3**  exp Decompression, Surgical/ (35766)  **4**  thorax.mp. or exp Thorax/ (61117)  **5**  3 and 4 (109)  **6**  1 or 2 or 5 (356)  **7**  tension pneumothorax.mp. or exp Pneumothorax/ (18980)  **8**  (tension adj4 pneumothorax).mp. [mp=title, book title, abstract, original title, name of substance word, subject heading word, floating sub-heading word, keyword heading word, organism supplementary concept word, protocol supplementary concept word, rare disease supplementary |
| --- |
| concept word, unique identifier, synonyms, population supplementary concept word, anatomy supplementary concept word] (1516)  **9**  7 or 8 (19005)  **10**  6 and 9 (132)  **11**  limit 10 to (english language and yr="2003 -Current") (121)  **12**  failure$.mp. (1080768)  **13**  10 and 12 (31)  **14**  limit 13 to (english language and yr="2003 -Current") (30) |
| **Database: Embase <1974 to 2024 April 30>**  **Search Strategy:**  **1**  needle decompression.mp. (386)  **2**  (needle adj2 decompression).mp. [mp=title, abstract, heading word, drug trade name, original title, device manufacturer, drug manufacturer, device trade name, keyword heading word, floating subheading word, candidate term word] (420)  **3**  exp needle/ and exp decompression/ (372)  **4**  exp decompression surgery/ (64751)  **5**  1 or 2 or 3 or 4 (65347)  **6**  tension pneumothorax.mp. or tension pneumothorax/ (2616)  **7**  exp pneumothorax/ or pneumothorax.mp. (57627)  **8**  (tension adj4 pneumothorax).mp. [mp=title, abstract, heading word, drug trade name, original title, device manufacturer, drug manufacturer, device trade name, keyword heading word, floating subheading word, candidate term word] (2677)  **9**  6 or 7 or 8 (57627)  **10**  5 and 9 (553)  **11**  limit 10 to (english language and yr="2003 -Current") (505)  **12**  failure$.mp. or exp failure analysis/ (1898651)  **13**  10 and 12 (134)  **14**  limit 13 to (english language and yr="2003 -Current") (128) |
| **Database: Web of Science <1970 to 2024 April 30>**  **Search Strategy:** |
| 1. "Needle Decompression" AND "Tension Pneumothorax" |
| 1. "Failure Rate" AND "Needle Decompression" AND "Tension Pneumothorax" |
| 1. "Tension Pneumothorax" AND "Treatment Outcomes" AND "Needle Decompression" |
| 1. "Pneumothorax Management" AND "Decompression Failure" |
| 1. "Clinical Efficacy" AND "Needle Decompression" AND "Pneumothorax" |
| 1. "Complications" AND "Needle Decompression" IN "Tension Pneumothorax" |
| 1. "Success Rate" AND "Needle Decompression" AND "Tension Pneumothorax" |
| 1. "Emergency Treatment" AND "Pneumothorax" AND "Decompression Techniques" |
| 1. "Thoracic Procedures" AND "Decompression Failure" AND "Pneumothorax" |
| 1. "Needle Decompression" AND "Pneumothorax" AND "Systematic Review" |

**Supplementary Table 1b:** Search strategy

| Embase <1974 to 2024 April 30> | |
| --- | --- |
|  |  |
| 1 | thorax/ or thorax wall/ or thorax injury/su, th or thorax drainage/ |
| 2 | (chest wall or thora*).ti,ab. |
| 3 | needle/ or (needle or catheter).ti,ab. |
| 4 | decompression/ or decompres*.ti,ab. |
| 5 | thoracostomy/ or thoracocentesis/ or (thoracostom* or thoracocentesis).ti,ab. |
| 6 | pneumothorax/su, th or tension pneumothorax/su, th or pneumothora*.ti,ab. |
| 7 | 1 or 2 |
| 8 | 3 or 4 or 5 |
| 9 | 6 and 7 and 8 |
| 10 | (exp animal/ or exp invertebrate/ or nonhuman/ or animal experiment/ or animal tissue/ or animal model/ or exp plant/ or exp fungus/) not (exp human/ or human tissue/) |
| 11 | 9 not 10 |

**Supplementary Table 2:** Data extraction

| **Basic Data** | - Characteristics of the study:  a.) first author, b.) title, c.) year of publication d.) journal’s name, quartile and publishing country e.) country of conduct, f.) study design and level of evidence, g.) quality of study, h.) sample size, i.) clinical setting - Characteristics of the included study participants:  a.) complete cases N, b.) age, c.) sex, d.) BMI, e.) injury severity score - Primary and secondary outcomes adjusted to our study - Risk of bias assessment - Heterogeneity |
| --- | --- |
| **Interventional studies** | - Needle length (cm) - Type of needle - Needle thoracostomy location - CWT (cm) measured with US (w./wo. pressure), CT, MRI, or other - Definition of success - Success rate of the intervention in % (w./wo. pressure) - Blunt or penetrating tension pneumothorax - Complications due to intervention |
| **Non-Interventional studies** | - CWT (cm) and type of measurement |

**Supplementary Table 3:** The 51 studies included in the meta-analysis.

| Number | Title | First Author | | Author Country | | Author Country | Year | | | Journal | | Journal Quartile | | Country of Publication |
| --- | --- | --- | --- | --- | --- | --- | --- | --- | --- | --- | --- | --- | --- | --- |
| 1 | A Retrospective Analysis of Needle Thoracostomies at a Tertiary Level 2 Trauma Center | Sarthak Parikh | | USA | | USA | 2024 | | | Cureus | | Q4 | | USA |
| 2 | Anterior versus lateral needle decompression of tension pneumothorax: comparison by computed tomography chest wall measurement | Leon D Sanchez | | USA | | USA | 2011 | | | Academic Emergency Medicine | | Q1 | | USA |
| 3 | Are needle decompressions for tension pneumothoraces being performed appropriately for appropriate indications? | Fernando Antonio Campelo Spencer Netto | | Canada | | Canada | 2008 | | | The American journal of emergency medicine | | Q1 | | USA |
| 4 | Association between three prehospital thoracic decompression techniques by physicians and complications: a retrospective, multicentre study in adults | Alan Garner | | Australia | | Australia | 2022 | | | European Journal of Trauma and Emergency Medicine | | Q3 | | Germany |
| 5 | Average Chest Wall Thickness at the Point of Needle Decompression in Thai Patients | Sivit Chanthawatthanarak | | Thailand | | Thailand | 2019 | | | Journal of the Medical Associateion of Thailand | | Q4 | | Thailand |
| 6 | Average chest wall thickness at two anatomic locations in trauma patients | Elizabeth Schroeder | | USA | | USA | 2013 | | | Injury | | Q2 | | England |
| 7 | Chest wall thickness in military personnel: implications for needle thoracentesis in tension pneumothorax | H Theodore Harcke | | USA | | USA | 2007 | | | Military Medicine | | Q4 | | UK |
| 8 | Clinical Characteristics of Patients Undergoing Needle Thoracostomy in a Canadian Helicopter Emergency Medical Service | Graham Newton | | Canada | | Canada | 2022 | | | Prehospital Emergency Care | | Q3 | | USA |
| 9 | Decompression of tension pneumothoraces in Asian trauma patients: greater success with lateral approach and longer catheter lengths based on computed tomography chest wall measurements | Serene Goh | Singapore | | Singapore | | | 2018 | European Journal of Trauma and Emergency Medicine | | Q3 | | Germany | |
| 10 | Determination of the appropriate catheter length and place for needle thoracostomy by using computed tomography scans of pneumothorax patients | Haldun Akoglu | | Turkey | | Turkey | 2013 | | | Injury | | Q2 | | England |
| 11 | Determination of the appropriate catheter length for needle thoracostomy by using computed tomography scans of trauma patients in Japan | Takeshi Yamagiwa | | Japan | | Japan | 2012 | | | Injury | | Q2 | | England |
| 12 | Emergent needle thoracostomy in prehospital trauma patients: a review of procedural execution through computed tomography scans | Michael M Neeki | | USA | | USA | 2021 | | | Trauma surgery and acute care open | | Q4 | | UK |
| 13 | Evaluation of 8.0-cm needle at the fourth anterior axillary line for needle chest decompression of tension pneumothorax | Samuel J Chang | | USA | | USA | 2014 | | | Journal of Trauma and Acute Care Surgery | | Q2 | | USA |
| 14 | Failure rate of prehospital chest decompression after severe thoracic trauma | Alexander Kaserer | | Switzerland | | Switzerland | 2017 | | | The American journal of emergency medicine | | Q1 | | USA |
| 15 | Failure Rate of Prehospital Needle Decompression for Tension Pneumothorax in Trauma Patients | Richard N Lesperance | | USA | | USA | 2018 | | | American Surgeon | | Q4 | | USA |
| 16 | Improvement in the prehospital recognition of tension pneumothorax: the effect of a change to paramedic guidelines and education | Kate Cantwell | | Australia | | Australia | 2014 | | | Injury | | Q2 | | England |
| 17 | Inadequate needle thoracostomy rate in the prehospital setting for presumed pneumothorax: an ultrasound study | Michael Blaivas | | USA | | USA | 2010 | | | Journal of ultrasound in medicine | | Q2 | | UK |
| 18 | Is routine tube thoracostomy necessary after prehospital needle decompression for tension pneumothorax? | Kathleen M Dominguez | | USA | | USA | 2013 | | | American journal of surgery | | Q1 | | USA |
| 19 | Measure of chest wall thickness in French soldiers: which technique to use for needle decompression of tension pneumothorax at the front? | Antoine Lamblin | | France | | France | 2014 | | | Military Medicine | | Q4 | | UK |
| 20 | Needle Decompression in Appalachia Do Obese Patients Need Longer Needles? | Thomas Edward Carter | | USA | | USA | 2013 | | | Western Journal of Emergency Medicine | | Q1 | | USA |
| 21 | Needle decompression of tension pneumothorax: Population-based epidemiologic approach to adequate needle length in healthy volunteers in Northeast Germany | Matthias Hecker | | Germany | | Germany | 2016 | | | Journal of Trauma and Acute Care Surgery | | Q2 | | USA |
| 22 | Needle thoracocentesis in tension pneumothorax: insufficient cannula length and potential failure | Simon Britten | | UK | | UK | 1996 | | | Injury | | Q2 | | England |
| 23 | Needle Thoracostomy for Patients with Prolonged Transport Times: A Case-control Study | Lori Weichenthal | | USA | | USA | 2015 | | | Prehospital and disaster medicine | | Q2 | | USA |
| 24 | Needle thoracostomy for tension pneumothorax: failure predicted by chest computed tomography | Robert L Stevens | | USA | | USA | 2009 | | | Prehospital Emergency Care | | Q3 | | USA |
| 25 | Needle thoracostomy for tension pneumothorax: the Israeli Defense Forces experience | Jacob Chen | | Israel | | Israel | 2015 | | | Canadian Journal of Surgery | | Q2 | | Canada |
| 26 | Needle thoracostomy in the prehospital setting | Marc Eckstein | | USA | | USA | 1998 | | | Prehospital Emergency Care | | Q3 | | USA |
| 27 | Needle Thoracostomy in the Prehospital Setting: A Retrospective Observational Study | Lori Weichenthal | | USA | | USA | 2016 | | | Prehospital Emergency Care | | Q3 | | USA |
| 28 | Needle thoracostomy in the treatment of a tension pneumothorax in trauma patients: what size needle? | Imme Zengerink | | The Netherland | | The Netherland | 2008 | | | Journal of Trauma and Acute Care Surgery | | Q2 | | USA |
| 29 | Needle thoracostomy may not be indicated in the trauma patient | Daniel C. Cullinane | | USA | | USA | 2001 | | | Injury | | Q2 | | England |
| 30 | Needle thoracostomy: Clinical effectiveness is improved using a longer angiocatheter | Johnathon M Aho | | USA | | USA | 2016 | | | The journal of trauma and acute care surgery. | | Q1 | | USA |
| 31 | Needle Thoracostomy: Does Changing Needle Length and Location Change Patient Outcome? | Lori A Weichenthal | | USA | | USA | 2018 | | | Prehospital and disaster medicine | | Q2 | | USA |
| 32 | Needle thoracostomy: implications of computed tomography chest wall thickness | Melissa L Givens | | USA | | USA | 2004 | | | ACADEMIC EMERGENCY MEDICINE | | Q1 | | USA |
| 33 | Optimal anatomical location for needle chest decompression for tension pneumothorax: A multicenter prospective cohort study | N Azizi | | The Netherland | | The Netherland | 2021 | | | Injury | | Q2 | | England |
| 34 | Paramedic use of needle thoracostomy in the prehospital environment | Keir J Warner | | USA | | USA | 2008 | | | Prehospital Emergency Care | | Q3 | | USA |
| 35 | Prehospital Decompression for Suspected Tension Pneumothorax | Holly Herron | | USA | | USA | 1994 | | | Air Medical Journal | | Q4 | | USA |
| 36 | Prehospital decompression of tension pneumothorax: Have we moved the needle? | Jordan Osterman | | USA | | USA | 2022 | | | American journal of surgery | | Q1 | | USA |
| 37 | Prehospital needle aspiration and tube thoracostomy in trauma victims: a six-year experience with aeromedical crews | Erik Barton | | USA | | USA | 1995 | | | Journal of Emergency Medicine | | Q3 | | USA |
| 38 | Prehospital needle thoracostomy: What are the indications and is a post-trauma center arrival chest tube required? | Benjamin Axtman | | USA | | USA | 2019 | | | American journal of surgery | | Q1 | | USA |
| 39 | Proper catheter selection for needle thoracostomy: a height and weight-based criteria | William F Powers | | USA | | USA | 2014 | | | Injury | | Q2 | | England |
| 40 | Radiologic Assessment of Potential Sites for Needle Decompression of a Tension Pneumothorax | David B Wax | | USA | | USA | 2007 | | | Anesthesia and analgesia | | Q1 | | USA |
| 41 | Radiologic Evaluation of Alternative Sites for Needle Decompression of Tension Pneumothorax | Kenji Inaba | | USA | | USA | 2012 | | | Archives of surgery | | Q1 | | USA |
| 42 | Risk of Harm in Needle Decompression for Tension Pneumothorax | Patrick Thompson | | USA | | USA | 2023 | | | Journal of special operations medicine | | Q4 | | USA |
| 43 | Risk values of weight and body mass index for chest wall thickness in patients requiring needle thoracostomy decompression. | Chia-Hung Hsu | | Taiwan | | Taiwan | 2020 | | | Emergency Medicine International | | Q4 | | UK |
| 44 | Safety and Risk Factors of Needle Thoracentesis Decompression in Tension Pneumothorax in Patients over 75 Years Old | Yanhu Wang | | China | | China | 2023 | | | Canadian respiratory journal | | Q4 | | UK |
| 45 | Sonographic evaluation of chest wall thickness in Chinese adults in Hong Kong: Should the updated (10th edition) Advance Trauma Life Support guidelines on preferred site of needle thoracocentesis in tension pneumothorax be adopted in the Asian population? | Stephanie Dorothy Pui-Ming Yu | | Hong Kong | | Hong Kong | 2020 | | | Trauma | | Q4 | | USA |
| 46 | The accuracy of chest wall thickness: To improve success rate of emergency needle thoracostomy. | Jatuporn Sirikun | | Thailand | | Thailand | 2017 | | | Journal of the Medical Associateion of Thailand | | Q4 | | Thailand |
| 47 | The safety and efficacy of prehospital needle and tube thoracostomy by aeromedical personnel | Daniel P Davis | | USA | | USA | 2005 | | | Prehospital emergency care. | | Q2 | | UK |
| 48 | Thoracic needle decompression for tension pneumothorax: clinical correlation with catheter length | Chad G Ball | | USA | | USA | 2010 | | | Canadian Journal of Surgery | | Q2 | | Canada |
| 49 | Ultrasound determination of chest wall thickness: implications for needle thoracostomy | Alan Robb McLean | | USA | | USA | 2011 | | | The American Journal of Emergency Medicine | | Q1 | | USA |
| 50 | Using Ultrasound to Determine Optimal Location for Needle Decompression of Tension Pneumothorax: A Pilot Study | Mathew Nelson | | USA | | USA | 2022 | | | Journal of Emergency Medicine | | Q3 | | USA |
| 51 | What Is the Optimal Device Length and Insertion Site for Needle Thoracostomy in UK Military Casualties? A Computed Tomography Study | Georgina Blenkinsop | | Germany | | Germany | 2015 | | | Journal of special operations medicine | | Q4 | | USA |

**Supplementary Table 4:** Chest wall thickness (Sex)

| Chest wall thickness in Males (cm) | Chest wall thickness in females (cm) |
| --- | --- |
| Average chest wall 2 MCL: 3.464(2.09-4.87) | Average chest wall 2 MCL: 3.414 (1.68-5.295) |
|  |  |
| Average chest wall 5 AAL: 2.540(2.6-4.56) | Average chest wall 5 AAL: 2.540(2.3-4.55) |
|  |  |
| Average chest wall 5 MAL: 3.213(2.13-5.46) | Average chest wall 5 MAL: 2.55(1.56-5.07) |

**Supplementary Table 5**: Mean Chest Wall Thickness Measurements (cm) Across Different Countries and Anatomical Regions

| **Mean Chest Wall Thickness 2 MCL Males** | **Mean Chest Wall Thickness 2 MCL Females** | **Weighted Mean Chest Wall Thickness 4MAL Males** | **Weighted Mean Chest Wall Thickness 4MAL Females** |
| --- | --- | --- | --- |
| France: 4.22 | France: 3.39 | France: 3.02 | France: 2.53 |
| Germany: 4.87 | Germany: 5.295 | Hong Kong: 2.36 | Hong Kong: 2.47 |
| Hong Kong: 2.67 | Hong Kong: 2.58 | USA: 5.521 | USA: 4.969 |
| Japan: 2.85 | Japan: 3.66 |  |  |
| Taiwan: 4.73 | Taiwan: 4.49 |  |  |
| Thailand: 3.0125 | Thailand: 3.3575 |  |  |
| The Netherland: 2.995 | The Netherland: 3.24 |  |  |
| Turkey: 3.795 | Turkey: 5.195 |  |  |
| USA: 3.4814 | USA: 2.4945 |  |  |
| ANOVA Test Results: | ANOVA Test Results: | ANOVA Test Results: | ANOVA Test Results: |
| F-value: 0.966 | F-value: 1.111 | F-value: 0.190 | F-value: 0.241 |
| P-value: 0.519 | P-value: 0.442 | P-value: 0.851 | P-value: 0.821 |
|  |  |  |  |
| **Mean Chest Wall Thickness 5 AAL Males** | **Mean Chest Wall Thickness 5 AAL Females** | **Mean Chest Wall Thickness 5 MAL Males** | **Mean Chest Wall Thickness 5 MAL Females** |
| China: 2.57 | China: 2.81 | China: 2.81 | Japan: 3.84 |
| Taiwan: 3.313 | Taiwan: 4.529 | Japan: 3.265 | Taiwan: 2.92 |
| The Netherland: 2.50 | The Netherland: 2.60 | USA: 3.663 | USA: 3.477 |
| USA: 3.45 | USA: 3.955 |  |  |
| ANOVA Test Results: | ANOVA Test Results: | ANOVA Test Results: | ANOVA Test Results: |
|  |  |  |  |
| F-value: 0.127 | F-value: 1.245 | F-value: 0.102 | F-value: 0.069 |
| P-value: 0.933 | P-value: 0.564 | P-value: 0.908 | P-value: 0.936 |

**Supplementary Table 6a:** Assessing the radiological failure rates (US/CT/MRI) of different needle lengths, at 2MCL

| Needle Length (cm) | Measurement Site | Number of Patients | Average Failure Rate (%) |
| --- | --- | --- | --- |
| 3 | 2 MCL | 54 | 57 |
| 4.4 | 2 MCL | 110 | 50 |
| 4.5 | 2 MCL | 63 | 20 |
| 4.6 | 2 MCL | 100 | 47.3 |
| 5 | 2 MCL | 172 | 11.3 |
| 5.1 | 2 MCL | 100 | 35.2 |
| 5.5 | 2 MCL | 63 | 2.5 |
| 6 | 2 MCL | 63 | 1 |
| 6.1 | 2 MCL | 2574 | 20 |
| 6.4 | 2 MCL | 100 | 21 |
| 6.5 | 2 MCL | 2574 | 15 |
| 6.9 | 2 MCL | 2574 | 10 |
| 7 | 2 MCL | 593 | 1.8 |
| 7.7 | 2 MCL | 2574 | 5 |
| 8 | 2 MCL | 84 | 5.95 |
| 9.3 | 2 MCL | 2574 | 1 |

**Supplementary Table 6b:** Assessing the radiological failure rates (US/CT/MRI) of different needle lengths, at 5MAL

| Needle Length (cm) | Measurement Site | Number of Patients | Average Failure Rate (%) |
| --- | --- | --- | --- |
| 4.5 | 5 MAL | 63 | 11 |
| 5 | 5 MAL | 63 | 0 |
| 5.5 | 5 MAL | 63 | 0 |
| 6.5 | 5 MAL | 63 | 0 |
| 8 | 5 MAL | 84 | 2.21 |

**Supplementary Table 6c:** Assessing the radiological failure rates (US/CT/MRI) of different needle lengths, at 5AAL

| Needle Length (cm) | Measurement Site | Number of Patients | Average Failure Rate (%) |
| --- | --- | --- | --- |
| 4.5 | 5 AAL | 390 | 6.15 |
| 5 | 5 AAL | 680 | 16.7 |
| 7 | 5 AAL | 84 | 20.24 |

**Supplementary Table 7:** Inselflow

| Management of Pneumothorax Flowchart |
| --- |
| Initial Assessment |
| Symptoms: Sudden chest pain, shortness of breath, tachypnea, cyanosis Physical Exam: Decreased breath sounds, hyper-resonance on percussion, tracheal deviation (in tension pneumothorax) |
| Diagnosis |
| Imaging (if stable): - Chest X-ray - Ultrasound (FAST exam) - CT scan (if available) Confirm Pneumothorax? |
| If Confirmed |
| Type of Pneumothorax? - Simple Pneumothorax: Observation or Chest Tube Insertion - Tension Pneumothorax: Immediate Needle Decompression |
| Needle Decompression |
| Needle: 7cm Needle  Site Selection: Right tension pneumothorax: 2nd ICS at MCL or 5th ICS at MAL  Left tension pneumothorax: 5th ICS at MAL Needle Selection and Needle Insertion Technique: Clean site with antiseptic Insert needle perpendicular to chest wall Advance needle until air escapes Secure needle/catheter |
| Monitor for Clinical Improvement |
| Signs of Improvement? Yes: Continue Monitoring & Chest Tube Insertion No: Reassess Needle Position or Consider Alternative Diagnosis |
| Definitive Management |
| Chest Tube Insertion for Definitive Treatment Post-Decompression Monitoring: Monitor Vital Signs: BP, HR, SpO2, Respiratory Rate Relief of Respiratory Distress Assess Hemodynamic Stability Check for Improvement in Oxygen Saturation Evaluate Normalization of Breath Sounds |
| Monitor for Complications |
| Identify Complications? Yes: Manage Complications: - Lung Injury - Vascular Injury  - Cardiac Injury  - Needle Displacement - Hemothorax No: Continue Routine Monitoring and Follow-Up Imaging |
